# {225}_*γ*_ habit planes in martensitic steels: from the PTMC to a continuous model

**DOI:** 10.1038/srep40938

**Published:** 2017-01-20

**Authors:** Annick P. Baur, Cyril Cayron, Roland E. Logé

**Affiliations:** 1 Laboratory of Thermomechanical Metallurgy, PX Group Chair, Ecole Polytechnique Fédérale de Lausanne, 2002 Neuchâtel, Switzerland.

## Abstract

Fine twinned microstructures with {225}_*γ*_ habit planes are commonly observed in martensitic steels. The present study shows that an equibalanced combination of twin-related variants associated to the Kurdjumov-Sachs orientation relationship is equivalent to the Bowles and Mackenzie’s version of the PTMC for this specific {225}_*γ*_ case. The distortion associated to the Kurdjumov-Sachs orientation relationship results from a continuous modeling of the FCC-BCC transformation. Thus, for the first time, an atomic path can be associated to the PTMC.

Martensitic transformation in steels has been widely studied for the last century and remains a major field of research. This solid-phase transformation exhibits some particular crystallographic features such as the presence of habit planes between austenite (*γ*) and martensite (*α*), established orientation relationships between the two phases and a specific shape change. Based on these characteristics, various models have been developed in order to describe and understand the transformation. In the mid 50’s, two independent groups, Bowles and Mackenzie^1^, and Weschler, Liebermann and Read^2^ developed a similar approach to explain the main features of the transformation. These two equivalent models gave rise to one of the most famous theory of phase transformation: the *Phenomenlogical Theory of Martensitic Crystallography* (PTMC).

Among all the observed habit planes of martensite, the {225}_*γ*_ family is one of the most studied, because of the difficulties to be explained by the PTMC. The {225}_*γ*_ habit planes are visible in different martensite morphologies, such as thin plate martensite and butterfly martensite. Martensite morphologies with {225}_*γ*_ habit planes have a common characteristic: an internal inhomogeneous structure showing a high density of twins.

The first model that offers an explanation for {225}_*γ*_ habit planes is due to Jaswon and Wheeler in ref. 3. They proposed a transformation mechanism where such a family of plane remains untilted during the transformation, but not fully invariant. Based on the same atomic correspondence between martensite and austenite, the Bowles and Mackenzie’s version of the PTMC is the first model that can account for the invariant {225}_*γ*_ habit planes. They were however criticized because an additional dilatation parameter was needed in the model. Many experiments have been performed in order to observe and measure this dilatation. They all concluded that this parameter do not differ substantially from unity^4^,^5^,^6^. The Bowles and Mackenzie’s explanation for these habit planes was abandoned and almost forgotten.

Several other models have then been developed avoiding the use of the dilatation parameter^7^,^8^,^9^,^10^. The current one, called the *double shear theory*, was proposed independently by Acton and Bevis^9^ and Ross and Crocker^10^. In this theory, a second *lattice invariant shear*, crystallographically independent from the first one, is added to the original PTMC theory. However, as wisely observed by Dunne and Wayman^11^: “It appears that the refined and indeed elegant generalization of the original theory, wherein a single shear is replaced by two, leads to improved but not completely satisfactory prediction for the {225}_*γ*_ case. It is further disappointing that the generalization, compared to the original theory, imparts a complexity which provides no selection rules for the initial choice of shear elements. The present theoretical situation is thus one of *modus vivendi*.”

Starting from *a priori* completely different hypothesis than those of PTMC, Cayron recently proposed a continuous model for the FCC-BCC transformation^12^. To derive the atomic path, he assumed that the atoms behave as hard spheres and the final orientation relationship is exactly Kurdjumov-Sachs. It turns out that he in fact rediscovered the Jaswon and Wheeler distortion and proposes a continuous atomic path for it.

By investigating the properties of the distortion, the present paper shows that an appropriate combination of twin-related variants of this model is exactly equivalent to the Bowles and Mackenzie modeling of the {225}_*γ*_. It solves therefore, for this particular case, the question of the atomic path that could not be given by the PTMC. The crystallographic features of the {225}_*γ*_ plate martensite, like the shape strain and the twinning system, are directly derived from the distortion using simple linear algebra concepts and are in total agreement with the Bowles and Mackenzie analysis. Furthermore, an answer is proposed to an old open question raised by Jaswon and Wheeler in the conclusion of their article, namely, “the reason for the choice of {225}_*γ*_ habit in preference to an octahedral habit, since both have been shown to satisfy the condition of undergoing no directional change”^3^.

## Results

### Distortion associated with the Kurdjumov-Sachs orientation relationship

Experimental observations show that the orientation relationship between austenite and martensite is often found to be the Kurdjumov-Sachs relationship^13^:


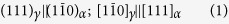


Considering atoms as hard spheres, the FCC-BCC martensitic transformation according to this particular orientation relationship can then be described by the total distortion matrix^12^:


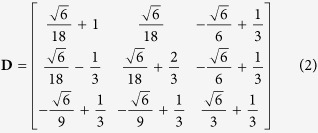


This matrix expressed according to the orientation relationship (1) is equivalent to the matrix 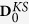 calculated in equation 32 of reference ref. 12 that was associated to the following equivalent orientation relationship: 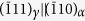 and [110]_*γ*_ ‖ [111]_*α*_. This matrix expresses the total distortion, from the initial to the final state. The continuous analytical expression associated to this distortion is given in reference ref. 12.

The FCC-BCC distortion **D** of equation (2) is not an invariant plane strain, but an invariant line strain, as only the 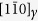 direction is undistorted during the transformation. By computing the distortion matrix in the reciprocal lattice D* = **D**^−*T*^ and extracting its eigenvectors, one can show that there are two planes which are not tilted by the distortion: (111)_*γ*_ and 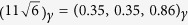. The (111)_*γ*_ plane is explicitly imposed to be untilted by the Kurdjumov-Sachs orientation relationship (1). On the contrary, the second plane 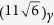 is not explicitly described by the Kurdjumov-Sachs orientation relationship and is of interest as it is oriented at 0.5° from the well-known (225)_*γ*_ habit plane.

### Variants of the distortion associated with the Kurdjumov-Sachs orientation relationship

Due to the orientation relationship and the crystalline symmetries, there exist 24 variants of Kurdjumov-Sachs. The distortion matrix 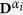 relative to each variant *α*_*i*_ is thus found using the symmetry properties of the FCC-BCC transformation^14^,^15^. In the present paper, we arbitrarily consider that the distortion matrix **D** presented in equation (2) is the distortion matrix relative to the first variant 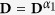. The absolute basis 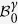 for expressing the transformation matrix is therefore equal to the basis relative to the first variant 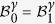. By convention, if the basis in which the vectors and matrix are expressed is different from the absolute basis 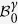. This basis appears explicitly in the notation as a right-down index. For example, a matrix **M** expressed in the *α*_*i*_ crystal is written 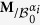. When the basis is the absolute one 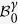, the basis is not specified in the index.

The transformation matrix relative to all the 24 variants in the absolute basis 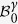 can be computed by using an appropriate change of basis:


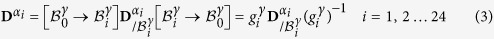


with 
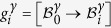
 a symmetry matrix of austenite.

For each variant *α*_*i*_, one arbitrarily chooses one 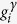 in its coset 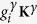, where 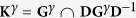 and **G**^*γ*^ is the point group of the austenite^15^. It is worth mentioning that in the particular case of FCC-BCC transformation, there is no distinction between orientational and distortional variants.

**Figure 1:**
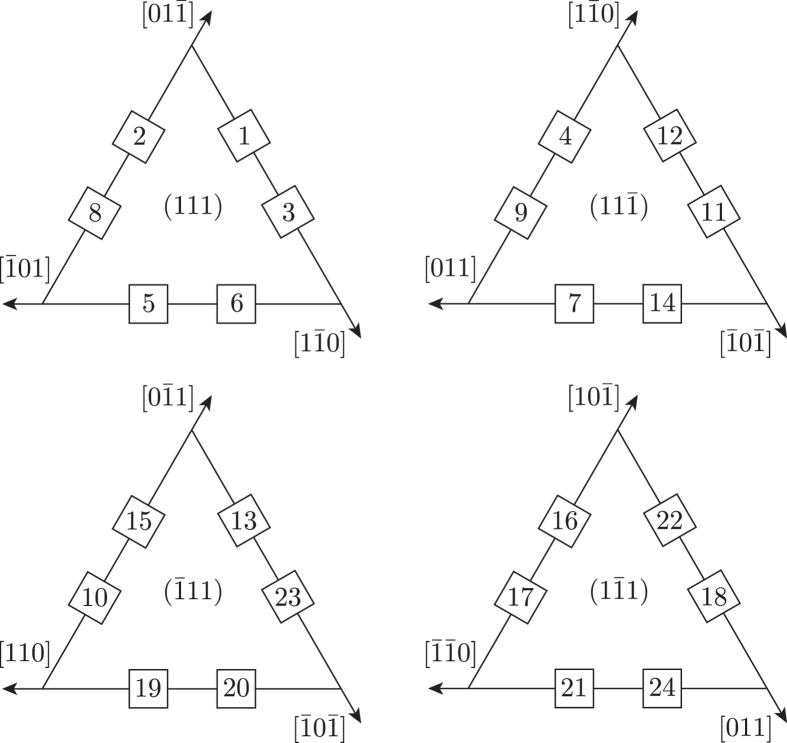
Common {111} planes and 〈110〉 directions of twin-related variants.

Among these 24 variants, there are 12 different pairs of twin-related variants. The twin-related variants of each pair have the particularity of sharing the same {111}_*γ*_ plane and the same 〈110〉_*γ*_ direction. This feature is illustrated in Fig. 1 for each particular {111}_*γ*_ plane. 3D representation of the crystallographic arrangements of the twin-related variants can be found in the supplementary material 3 of reference ref. 16.

In addition, it can be noted that the transformation matrix corresponding to each variant of these pairs leaves the same plane 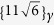 untilted. Table 1 summarizes the crystallographic nature of the pairs, indicating which planes are untilted and which direction is undistorted during the transformation. This table can also be read to identify the proper orientation relationship for each variant, this orientation relationship being defined by the invariant direction and the untitled plane of type {111}_*γ*_.

The twin-related variants of each pair can be expressed in the same *semi*-*eigenbasis* 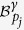 for *j* = 1, ..., 12 defined by the common 〈110〉_*γ*_ invariant line, the normal to the common 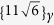 untilted plane and the cross product of these two vectors.

The terminology of *semi*-*eigenbasis* is used here in opposition to the classical eigenbasis. Indeed as the distortion associated with the Kurdjumov-Sachs orientation relationship has only one invariant line, it is not diagonalizable, and thus cannot be expressed in an eigenbasis^12^. In the rest of the paper, the mathematical development will be performed explicitly only for the pair *p*_1_ of variants *α*_1_ and *α*_3_, but the same calculation is applicable to all pairs *p*_*j*_. The transformation matrices of the twin-related variants *α*_1_ and *α*_3_ expressed in their common semi-eigenbasis are then:


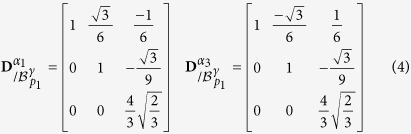


If expressed in their appropriate semi-eigenbasis 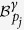, the distortion matrices of each of the twin-related variants of every pairs *p*_*j*_ are equal to the ones presented in equation (4).

### The Kurdjumov-Sachs invariant plane strain

By inspection, it exists only one possibility to achieve an invariant plane strain (IPS) from a linear combination of these two matrices, and it is a mixture with 1:1 volume ratio of each of the twin-related variants,


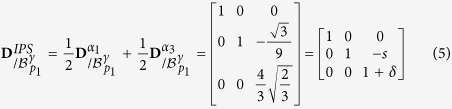


This matrix shows that a fine combination of alternating twin-related variants can produce a shape strain which is an IPS, having the 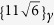 as invariant plane. The invariant plane of the average distortion **D**^*IPS*^ is disoriented by 0.5° from the {225}_*γ*_. The magnitude of the shape shear is 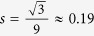 and the dilatation normal to the habit plane is 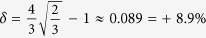. It should be noted that for twin-related variants pairs, a linear combination satisfy the volume conservation: 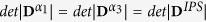.

### Interface between the twin-related variants

To complete the crystallographic study of the {225}_*γ*_ habit planes one needs to verify the geometrical compatibility of the transformation at the interface between the two twin-related variants. In other words, it is necessary that the interface plane between each twin-related variants is transformed in the same way by both variant. Mathematically, it consists in searching two non-collinear vectors **v** ∈ {**v**_1_, **v**_2_} such that for each of them^17^,^18^. 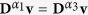. This condition is equivalent to calculating the Kernel of 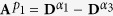:


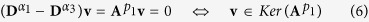


The computation shows that two non-collinear vectors 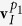 and 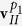 belong to the Kernel. Together they define the interface plane between the two twin-related variants. The normal 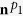 to this interface plane is simply found by calculating a cross-product, 
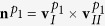
. Such Kernel can be computed for each pair *p*_*j*_ of twin-related variants.

It is usual to express the interface between the two variants in their own basis 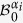. The distortion of the planes, expressed in the *α*_*i*_ basis 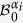, is given by the correspondence matrix for each *i* = 1, 2, ... 24 in the reciprocal lattice 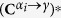. The interface plane between the twin-related variant is then:


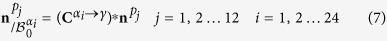


The results of the predicted interface between two twin-related variants are reported in Table 2 for each pair *p*_*j*_ of twins. Six different interfaces belonging to the {110}_*γ*_ planes family have been found. According to equation (7), they correspond to {112}_*α*_.

### Equivalence with Bowles & Mackenzie model

In the Bowles and Mackenzie’s paper *The crystallography of martensite transformations III*^1^, the shape deformation associated to their prediction is expressed in the basis 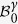 as follows,


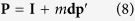


where 
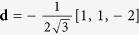
 and 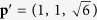 and 
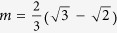
.

This transformation matrix can be expressed in its proper *semi*-*eigenbasis* in the same manner that has been used previously for the twin-related variant. It takes a form that is exactly the same as the Kurdjumov-Sachs invariant plane strain **D**^*IPS*^ produced by the composition of twin-related variants.


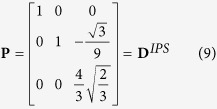


Consequently, the Bowles and Mackenzie’s version of the PTMC allows the prediction of the same 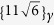 planes as the ones that are shown to stay invariant by an appropriate combination of twin-related variants.

## Discussion

It has been shown that an heterogenous structure of infinitely small twin-related variants in equal proportion leaves a plane completely invariant. This plane is a 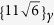 plane disoriented from the well-known {225}_*γ*_ plane by only 0.5°. The macroscopic shape deformation resulting from such a combination of variants, each of these variants undergoing an invariant line strain, is exactly an invariant plane strain.

**Figure 2:**
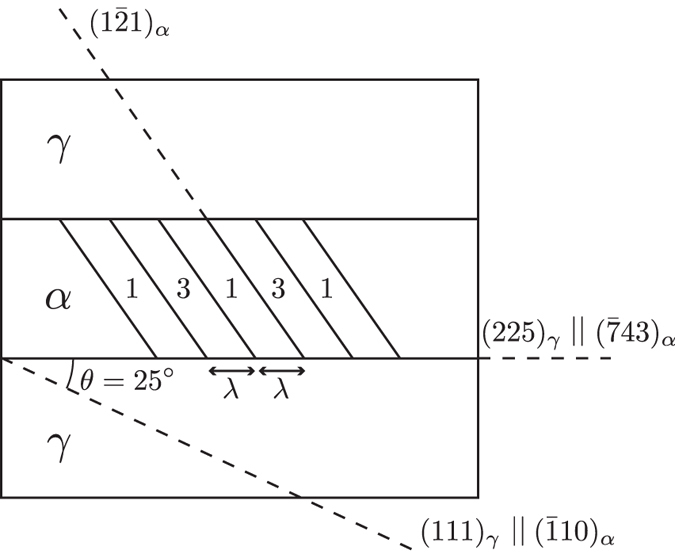
Schematic representation of (225)_*γ*_ thin plate of martensite. Cross-sectional view normal to 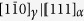.

Figure 2 schematically represents a (225)_*γ*_ thin plate of martensite according to the present results.

The thin plate martensite showing a high density of alternate twin-related variants *α*_1_ and *α*_3_ is represented surrounded by the austenitic matrix. The crystallographic orientations of the *γ* and *α* phases are defined thanks to the dashed lines representing particular planes. The trace of the (225)_*γ*_ predicted habit plane is also shown. This plane corresponds to the 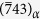 in the martensite phase. In the present study, all twelve habit planes predicted by the Kurdjumov-Sachs invariant plane strain **D**^*IPS*^ are the {225}_*γ*_ planes at 25° from the {111}_*γ*_ planes involved in the Kurdjumov-Sachs relationship. This particular feature is in full agreement with the experimental studies of Shimizu, Oka and Wayman on Fe-Cr-C alloy^19^. Each pair *p*_*j*_ of twin-related variants is then associated unequivocally with one of theses twelve {225}_*γ*_ habit planes. The proportion *λ* of each of the twin-related variants *α*_1_ and *α*_3_ that is needed to produce the invariant plane strain is unique and the ratio is found to be 1:1. This result corresponds to the value experimentally deduced by Kelly and Nutting^20^ when they studied the martensitic transformation in carbon steels. It is also the same proportion that is considered in the Bowles and Mackenzie’s model for this specific habit plane.

The present study also shows that the interface plane between two Kurdjumov-Sachs twin-related variants is unique, as there are exactly two non-collinar vectors in the Kernel computed in equation (6). The results suggest that the martensite twins are not created from mechanical twinning, but are due to a particular association of variants, these variants being twin-related. We conclude that a local variant selection occurs to accommodate the phase transformation on the habit plane. Away from the habit plane, however, the transformation might need additional mechanisms, like plasticity by dislocations gliding to accommodate the shape strain related to the transformation. Such additional mechanisms can be observed in {225}_*γ*_ habit planes martensitic steels^21^.

The shape strain **D**^*IPS*^ associated with an equibalanced combination of twin-related variants can be clearly identified in equation (5). This shape strain is exactly the same as the one predicted by the PTMC and computed in equation (9). The calculated shear is 0.19 and the dilatation normal to the habit plane is +8.9%. The shear strain is in good agreement with the magnitude experimentally measured in ferrous martensite which varies between 0.18^22^ and 0.22^23^. However, the dilatation normal to the habit plane is overestimated. Indeed, the dilatation reported in the literature is about +3%^23^. This discrepancy was expected because the atoms were assumed to be hard spheres of constant radius, whereas a slight decrease of the atomic size (few %) is observed during the transformation. This atomic size change is not captured by our model such that we overestimated the volume change associated to the FCC-BCC transformation, and hence the dilatation normal to the habit plane as well.

The hard-sphere assumption is done in both the Bowles and Mackenzie’s PTMC model for the {225}_*γ*_ case and our model. Indeed, for this specific habit plane, the PTMC requires the dilatation parameter to be different from 1, such that the 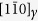 and the [111]_*α*_ atomic rows match. As commented by Bowles, this dilatation is exactly equivalent to an hard sphere modeling of the atoms (ref. 1, part III). In both models, the matching of the 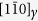 and the [111]_*α*_, and the exact Kurdjumov-Sachs orientation relationship are required to let the (225)_*γ*_ completely invariant. To compensate the natural decrease of the atom size and the consequent atomic mismatch along the 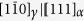 closed-pack direction, accommodation mechanisms by dislocations in the austenite need to be considered. Back in the 70’s, for example, Bowles envisaged multiple {111}_*γ*_ slip systems to preserve an invariant habit plane^24^. More recently, Stanford and Dunne used similar arguments to explain the austenite/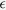-martensite interface in Fe-Mn-Si alloys^25^. The Bowles and Mackenzie’s dilatation parameter was controversial and so is also the hard sphere modeling of the atoms. However, this approach has the advantage of allowing the description of the atomic trajectories. In this respect, using the hard-sphere model may allow more significant insights into the effective transformation mechanism than the consideration of artificial double shear systems, mentioned in the introduction.

It is remarkable that both the atomistic and the phenomenological modeling lead to the same results. In fact, even though these approaches are *a priori* based on opposite starting hypothesis, they share one common assumption: the atomic correspondence between the austenite and the martensite. The Bowles and Mackenzie model is historically based on the observation of the macroscopic shape strain associated with the transformation. An initial guess on the lattice invariant shear is required and the orientation relationship can then be derived. It only deals with the initial and the final states, but allows to cover a broad range of transformation, morphologies and habit planes. On the contrary, in the model proposed by Cayron, one assumes the final orientation relationship and imposes a steric condition on the atomic trajectories, by the mean of the hard sphere assumption. A precise atomic path can thus be defined for the transformation. The shape strain and the twinning system are then directly derived from the model with simple calculations. The predicted twinning system corresponds exactly to the lattice invariant shear assumed in the PTMC and experimentally observed^19^, which confirms the equivalence of the two models in the {225}_*γ*_ case.

In their original papers, Bowles and Mackenzie emphasize the phenomenological nature of their theory. As reformulated by Dunne^24^, they stressed that the theory provides “a framework that any proposed transformation mechanism must satisfy”. The distortion associated to Kurdjumov-Sachs orientation relationship is shown to fit perfectly in this framework, for this particular {225}_*γ*_ case. Our model is thus a step toward a complete mechanistic representation of the transformation. In this regard, it might be noted that the mathematical approach used in this paper has also been successfully applied for {557}_*γ*_ habit planes in dislocated martensite^26^.

**Figure 3:**
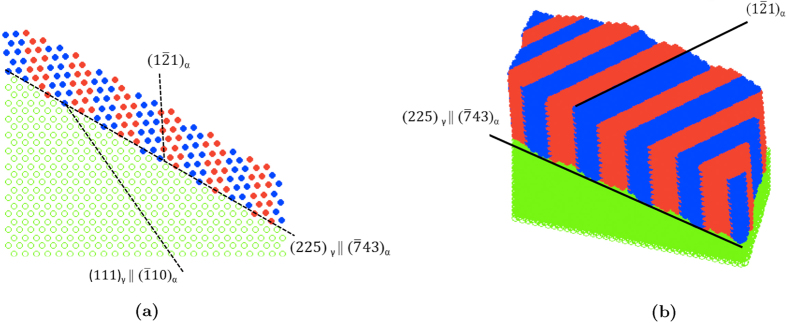
Illustrations of the (225)_*γ*_ thin plate: (**a**) projection along 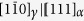 and (**b**) 3D view. Green dots: iron atoms in austenite. Blue and red dots: iron atoms in martensitic twin-related variants *α*_1_ and *α*_3_.

**Figure 4:**
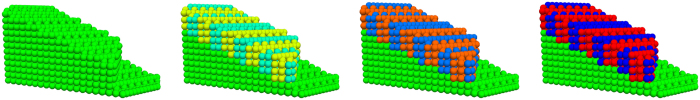
Snapshots of the (225)_*γ*_ thin plate formation. Green dots: iron atoms in austenite. Blue and red dots: iron atoms in martensitic twin-related variants *α*_1_ and *α*_3_.

The present study explains the invariant nature of the {225}_*γ*_ habit planes thanks to fundamental, but rather abstract, linear algebra concepts. So, in order to visualize the crystallography of {225}_*γ*_ thin plates, some computer simulations have been performed at the atomic scale. These simulations consist in computing the two final twin-related BCC lattices within their parent austenitic matrix. The computation of the transformation is based on the matrices 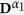 and 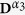 from equation (3). Only the iron atoms are considered and illustrated in Fig. 3. Figure 3(a), analogous to the schematic Fig. 2, shows the accurate atomic positions. It illustrates clearly the Kurdjumov-Sachs orientation relationship, 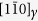 being parallel to [111]_*α*_ direction, and (111)_*γ*_ plane parallel to 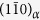. All the crystallographic features presented schematically in Fig. 2 are also illustrated here. Figure 3(b) shows a tridimensional view of the simulated {225}_*γ*_ plate. The proposed modeling of the {225}_*γ*_ habit plane martensite is based on an atomic description of the FCC-BCC phase transformation^12^. A movie of the simulated {225}_*γ*_ thin plate of martensite was computed. It is available in the supplementary material. Snapshots of this film are presented in Fig. 4.

**Figure 5:**
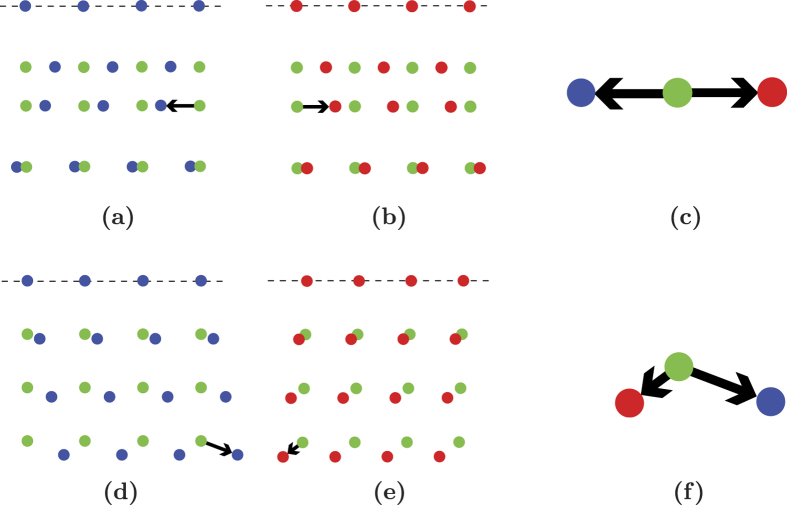
Comparison between the atomic displacements in the 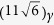 plane (**a**–**c**) and in the (111)_*γ*_ plane (**d**–**f**). Green dots: iron atoms in austenite. Blue and red dots: iron atoms in martensitic twin-related variants *α*_1_ and *α*_3_.

As previously mentioned, the distortion associated with the Kurdjumov-Sachs orientation relationship leaves two families of plane untilted, {111}_*γ*_ and 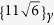. However, it is usually the second family of planes which is experimentally observed. As mentioned in the introduction and already questioned by Jaswon and Wheeler^3^, a major question is then to understand why. The present approach proposes a clear answer, illustrated in Fig. 5. In the distortion, each of the untilted planes is deformed within the plane, as the spacing between the atoms in this plane changes during the transformation^12^. The matrices computed in equation (4) show that for the (225)_*γ*_ habit plane the atoms are displaced in opposite directions for each twin of a given pair of twin-related variants such that the average displacement on this plane is zero. This average cancellation is illustrated in Fig. 5(a) and (b). They show the atomic positions in a 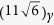 habit plane for both variants *α*_1_ and *α*_3_. The 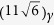 plane being irrational, it cannot form a 2D crystallographic lattice. Therefore, in the simulations, this plane is defined by all the atomic positions **u** such that: 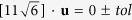, where *tol* is a tolerance factor equal to 0.05. Black arrows are sketched on figure in order to better visualize the atomic displacements. The invariant 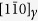 direction, where the atoms positions in austenite and in martensite match is noted on the picture with a dashed horizontal line. To be compared with Fig. 5(a) and (b), Fig. 5(d) and (e) show the atomic positions after transformation for each of the twin-related variants *α*_1_ and *α*_3_ in the (111)_*γ*_ plane. Figure 5(c) and (f) offer detailed views of the displacements for each variant in the (225)_*γ*_ plane and in the (111)_*γ*_ plane. Figure 5(c) illustrates the average cancellation of the displacements. On the contrary, as showed in Fig. 5(f), in the (111)_*γ*_ plane, there is a displacement on the vertical axis that goes in the same direction for both variants. Indeed, the red and blue dots both shift in a direction 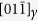 relatively to the initial FCC lattice in the same manner. Such a displacement cannot be cancelled by any combination of these two variants. This result can also be proved mathematically by applying an analogous approach for (111)_*γ*_ as the one we used to show the invariant nature of (225)_*γ*_ planes. We notice that all the volume change intrinsic to the FCC-BCC transformation occurs by the distortion of the (111)_*γ*_ plane. Therefore {111}_*γ*_ planes cannot be invariant during the transformation.

In conclusion, this study shows that a fine alternate structure of Kurdjumov-Sachs twin-related variants in equal proportion creates a macroscopic invariant plane strain having 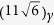 as invariant plane, lying at 0.5° from the observed (225)_*γ*_ plane. The shape strain resulting from this combination of twin-related variants consists in a shear of magnitude 0.19 parallel to the habit plane and a dilatation normal to the habit plane of +8.9%. This shape strain corresponds exactly to the result of the Bowles and Mackenzie’s version of the PTMC. For this special (225)_*γ*_ case, the two models are shown to be equivalent. We also demonstrated that because of the geometrical compatibility at the interface, Kurdjumov-Sachs twin-related variants share an interface plane of type {112}_*α*_, which corresponds to the twinning system that is assumed in the PTMC.

In this specific case, a continuous atomic displacement can be associated to the original Bowles and Mackenzie’s model, offering, for the first time, a mechanistic dimension to a theory which up to now was phenomenological.

## Methods

### Mathematical notations and conventions

The mathematical notations and conventions used in the present paper are briefly presented here. The vectors are column vectors and are written in small bold letters. The matrices are written in bold capital letters. A vector **v** is transformed by a matrix **M** as follows:


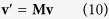


The coordinate-transformation matrix between two basis 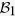 and 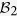 is noted 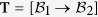 and is defined such that its columns are the vectors of the basis 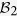 expressed in the basis 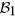:


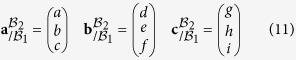


One has,


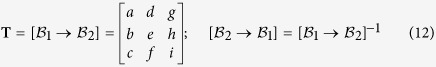


The vector **v** and the matrix **M** expressed in 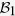, noted 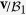 and 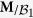 are, then, respectively expressed in 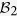 by:


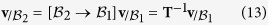



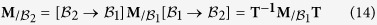


### Semi-eigenbasis of twin-related variants

The distortion matrices of variants *α*_1_ and *α*_3_ forming the first pair *p*_1_ are expressed in their common semi-eigenbasis, using the following coordinates-transformation matrix:


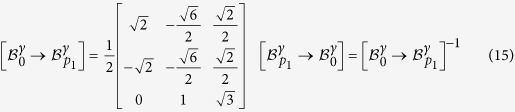


The distortion matrices 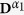 and 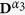 of each twin-related variant *α*_1_ and *α*_3_ can then be expressed in the common semi-eigenbasis.


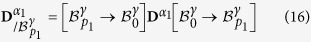



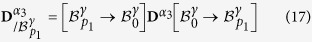


### Computation of the correspondence matrix

The matrix that transforms the coordinates of each crystal *α*_*i*_ into the coordinates of the crystal *γ* is 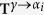
14:


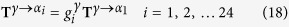


where 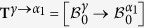. 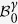 and 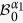 are formed by the usual crystallographic vectors of the Bravais lattice of the *γ* and *α*_1_ crystals, respectively.

The correspondence matrix 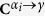 allows the computation of the image by the distortion 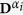 of any vector in the *γ* crystal expressed in the basis 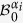 of the product crystal *α*_*i*_.

Indeed, the image of any vector 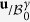 by the distortion is given by:


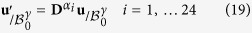


To express the transformed vector 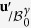 in the 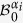 basis one needs the transformation matrix presented in equation (18):


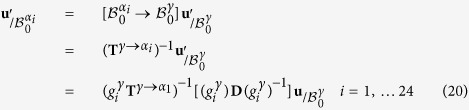


where 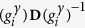 is the distortion matrix of variant *α*_*i*_ expressed in the absolute basis 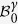, as computed in equation (3).

Based on equation (20), the correspondence matrix is then:


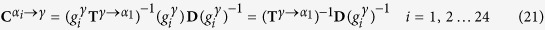


Considering the distortion associated with the Kurdjumov-Sachs orientation relationship presented in relation (1) and the present coordinate-transformation matrix, the correspondence matrix 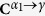 is:


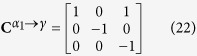


And the correspondence matrix in the reciprocal lattice is then:


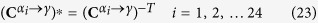


## Additional Information

**How to cite this article**: Baur, A. P. *et al*. {225}*_γ_* habit planes in martensitic steels: from the PTMC to a continuous model. *Sci. Rep.* **7**, 40938; doi: 10.1038/srep40938 (2017).

**Publisher's note:** Springer Nature remains neutral with regard to jurisdictional claims in published maps and institutional affiliations.

## Supplementary Material

Supplementary Video 1

Supplementary Information

## Figures and Tables

**Table 1:** Crystallographic features of the FCC-BCC transformation of twin-related variants.

Variants	*α*_1_	*α*_2_	*α*_4_	*α*_5_	*α*_7_	*α*_10_	*α*_11_	*α*_13_	*α*_16_	*α*_18_	*α*_19_	*α*_21_
	*α*_3_	*α*_8_	*α*_9_	*α*_6_	*α*_14_	*α*_15_	*α*_12_	*α*_23_	*α*_17_	*α*_22_	*α*_20_	*α*_24_
Pairs	*p*_1_	*p*_2_	*p*_3_	*p*_4_	*p*_5_	*p*_6_	*p*_7_	*p*_8_	*p*_9_	*p*_10_	*p*_11_	*p*_12_
Invariant direction	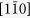	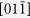	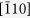	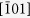	[011]	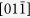	[101]	[101]	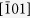	[011]	[110]	[110]
Untilted planes	(111) 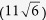	(111) 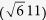	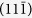 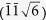	(111) 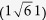	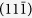 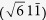	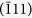 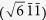	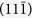 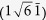	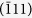 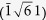	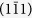 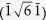	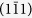 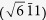	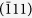 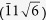	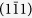 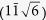

**Table 2:** Crystallographic features of the interface between twin-related variants.

Variants		*α*_1_	*α*_2_	*α*_4_	*α*_5_	*α*_7_	*α*_10_	*α*_11_	*α*_13_	*α*_16_	*α*_18_	*α*_19_	*α*_21_
		*α*_3_	*α*_8_	*α*_9_	*α*_6_	*α*_14_	*α*_15_	*α*_12_	*α*_23_	*α*_17_	*α*_22_	*α*_20_	*α*_24_
Pairs		*p*_1_	*p*_2_	*p*_3_	*p*_4_	*p*_5_	*p*_6_	*p*_7_	*p*_8_	*p*_9_	*p*_10_	*p*_11_	*p*_12_
Interface plane	in 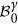	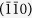	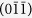	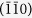	(101)	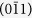	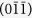	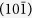	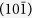	(101)	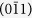	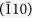	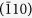
	in 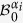	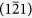	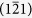	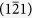	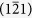	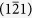	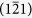	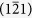	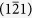	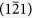	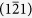	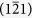	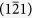
