# Supplementary material for: {225}γ habit planes in martensitic steels: from the PTMC to a continuous model
Source: Sci Rep. 2017 Jan 20;7:40938. doi: 10.1038/srep40938 (PMC5247700; doi:10.1038/srep40938)
Supplement: Supplementary Information [file srep40938-s2.pdf]

$\{225\}_\gamma$ habit planes in martensitic steels: from the PTMC to a continuous model.

Annick P. Baur, Cyril Cayron and Roland E. Logé

Legend of the supplementary information file

This video shows the formation of a $(225)_\gamma$ thin plate of martensite according to our continuous model. The green dots represent the iron atoms in austenite and the blue and red dots represent the iron atoms in the martensitic twin-related variants $\alpha_1$and α_3.
